# Breakthrough infections due to SARS-CoV-2 Delta variant: relation to humoral and cellular vaccine responses

**DOI:** 10.3389/fimmu.2023.1145652

**Published:** 2023-03-30

**Authors:** Matthieu Buscot, Marion Cremoni, Daisy Graça, Vesna Brglez, Johan Courjon, Jonathan Allouche, Maxime Teisseyre, Laurent Boyer, Jérôme Barrière, Emmanuel Chamorey, Michel Carles, Barbara Seitz-Polski

**Affiliations:** ^1^ Infectious Diseases Department, Nice University Hospital, Nice, France; ^2^ Immunology Laboratory, Archet 1 Hospital, Nice University Hospital, Nice, France; ^3^ Clinical Research Unit Côte d’Azur (UR2CA), Côte d’Azur University, Nice, France; ^4^ Mediterranean Center for Molecular Medicine (C3M), Côte d’Azur University, Nice, France; ^5^ Department of Oncology, Clinique St Jean, Cagnes sur Mer, France; ^6^ Department of Biostatistics, Centre Antoine Lacassagne, Nice, France

**Keywords:** COVID-19, breakthrough infections, vaccination, humoral response, cellular response

## Abstract

**Introduction:**

COVID-19 vaccines are expected to provide effective protection. However, emerging strains can cause breakthrough infection in vaccinated individuals. The immune response of vaccinated individuals who have experienced breakthrough infection is still poorly understood.

**Methods:**

Here, we studied the humoral and cellular immune responses of fully vaccinated individuals who subsequently experienced breakthrough infection due to the Delta variant of SARS-CoV-2 and correlated them with the severity of the disease.

**Results:**

In this study, an effective humoral response alone was not sufficient to induce effective immune protection against severe breakthrough infection, which also required effective cell-mediated immunity to SARS-CoV-2. Patients who did not require oxygen had significantly higher specific (p=0.021) and nonspecific (p=0.004) cellular responses to SARS-CoV-2 at the onset of infection than those who progressed to a severe form.

**Discussion:**

Knowing both humoral and cellular immune response could allow to adapt preventive strategy, by better selecting patients who would benefit from additional vaccine boosters.

**Trial registration numbers:**

https://clinicaltrials.gov, identifier NCT04355351; https://clinicaltrials.gov, identifier NCT04429594.

## Introduction

Coronavirus disease 2019 (COVID-19) vaccines are expected to provide an effective protection, since being the most appropriate preventive approach to SARS-CoV-2 pandemic (1–6). Emerging strains, such as the Delta (B.1.617.2) or Omicron (B1.1.529) variants may lead to infection in previously vaccinated individuals (also called “breakthrough infection”) (7–11), warranting additional preventive measures and booster vaccine doses, especially in the elderly, immunocompromised patients, and those with multiple comorbidities (12, 13). As pointed out elsewhere, the definition of a breakthrough infection is questionable, because a mild (or asymptomatic) clinical presentation has limited public health implications (14). Breakthrough infection rate has been initially evaluated of approximately 5% in fully vaccinated individuals (15, 16).

Vaccine efficacy rates in real-life observational settings are noticeable but partly time-dependent, referring to a waning immunity and is also compromised in immunosuppressed patients (4, 8, 17–19). From an immunological point of view, the occurrence of breakthrough infection in vaccinated individuals depends on two mechanisms: (i) the lack of induction of a neutralising immune response with the establishment of a specific B and T response or (ii) the lack of induction of immune memory response which will result in a loss of specific B and T response over time (20). The immune response to a viral agent, including SARS-CoV-2, or to mRNA-vaccine, involves both the innate and adaptative responses. Innate immunity induced by Toll-like receptors 3 (TLR3) ant TLR7/8 signalling activates effector cells to mediate viral clearance, induces inflammation through secretion of proinflammatory cytokines (e.g., IL-6 and IL-1β), produces antiviral cytokines and stimulates adaptative immune response by activating antigen-specific T cells. Type I and II interferons (IFN), i.e., IFN-α/β and IFN-γ, respectively, are the first line cytokines that fight viral infections. CD4 T cells will then be able to activate specific cytotoxic T cells and activate B cells that will differentiate into plasma cells capable of producing neutralising antibodies. Previous data demonstrated the importance of both humoral and cellular responses, even more in immunocompromised patients (21). This response will allow the establishment of a contingent of memory cells.

Although widely studied at the beginning of the pandemic, prior to vaccination, the immune response of vaccinated individuals that experienced breakthrough infection is still poorly understood. It is now commonly accepted that a sufficient level of anti-spike and anti-RBD antibodies after vaccination leads to a reduced risk of symptomatic breakthrough infection (22) even if this cannot be inferred to Omicron. However, the data regarding the humoral response of individuals undergoing breakthrough infection are conflicting (23–25). As for the cellular response of patients with breakthrough infection, very few data are available to date. Interestingly, Bastard et al. showed that despite vaccination and the presence of circulating antibodies capable of neutralizing SARS-CoV-2, type I IFN-neutralizing autoantibodies may account for a significant proportion of COVID-19 hypoxemic pneumonia cases (26). To our knowledge, no study has investigated type II IFN in the context of breakthrough infection. Indeed, if type I IFN is a component of innate immunity, type II IFN is involved in both innate and adaptative immune responses: IFN-γ is produced by natural killer cells and macrophages, effector cells in innate immunity, as well as by CD4+ T cells of the Th1 type and CD8+ T cells that participate in the adaptative response.

Thus, the objective of this study was to assess the combined humoral and cellular immune responses of fully vaccinated individuals that further experienced breakthrough infection of various severity levels, during the Delta variant wave. Our hypothesis is that identifying predictive criteria of severity at an early stage of SARS-CoV-2 infection should allow to strengthen early therapeutic strategies. Overall, we will propose a new approach able to better predict the risk of severe infection as well as the need (or not) for additional boosters.

## Methods

### Study design, participants, and data collection

We performed a prospective monocentric longitudinal and ancillary study at the Nice University Hospital, France. The participants were included from two cohorts: (i) patients recruited during an infectious diseases or emergency room consultation following COVID-19 symptoms, or as contact of a diagnosed COVID-19 case (CovImmune 1 study, NCT04355351); (ii) participants monitored periodically since July 2020 as part of an epidemiological study in the context of COVID-19 (CovImmune 2 study, NCT04429594) and developing a SARS-CoV-2 infection. Patients were eligible for inclusion if: (i) they received a complete vaccination regimen, i.e. at least two doses for mRNA-vaccine (either BNT162b2 or mRNA-1273) or ChAdOx1-S recombinant vaccine, or one dose of the Ad26.COV2.S vaccine; (ii) they developed a SARS-CoV-2 infection, symptomatic or not, in the aftermath of the vaccination; (iii) SARS-CoV-2 infection was confirmed by a nasopharyngeal PCR or an antigenic test; (iv) the last vaccine dose was injected at least ten days before the first symptoms (symptomatic cases) or the positive SARS-CoV-2 test for contact-cases (asymptomatic cases); (v) they were infected between August 2021 and January 2022, assuming that the COVID-19 cases for which the strain was not determined were infected with the Delta variant, given its predominance in our region during this period. Demographic, clinical, biological, and outcome data were collected by the study investigators and centralized in a database. If necessary, a clinical research assistant contacted participants by telephone to complete demographic and clinical data so that none of these data were missing. The study protocol complies with the principles of the Declaration of Helsinki and was approved by the *Comité de Protection des Personnes Sud-Ouest et Outre-Mer* institutional review board. Written inform consent was obtained from all study participants.

### Humoral responses

#### SARS-CoV-2-specific IgG antibodies

Serological tests for anti-SARS-CoV-2 IgG antibodies were performed on serum using a commercially available enzyme-linked immunosorbent assay (ELISA) which used the S1-domain of the spike protein of SARS-CoV-2 as the antigen (Euroimmun AG, Lübeck, Germany, #EI 2606-9601 G). They were run on IF Sprinter IFT/ELISA (Euroimmun) according to the manufacturer’s protocol. The SARS-CoV-2 IgG antibody titers were expressed in binding antibody units (BAU)/mL (WHO standard unit).

#### SARS-CoV-2 neutralization assay

The level of SARS-CoV-2 neutralizing antibodies in patient serum was estimated by a binding inhibition assay. The V-PLEX SARS-CoV-2 Panel 17 (ACE2) Kit (Meso Scale Discovery) was used to measure the ability of patient serum to inhibit binding of the SARS-CoV-2 Spike protein to the soluble angiotensin-converting enzyme 2 (ACE2) receptor. Signals were then converted to percentage according to the formula provided by the manufacturer:


$$\%\;inhibition=\;\left(1-\frac{average\;sample\;ECL\;signal}{average\;ECL\;signal\;of\;calibrator\;8\;\left(diluent\;only\right)}\right)\cdot 100$$

To note, antibody tests were performed prior to preventive anti-SARS-CoV-2 monoclonal antibodies injection. Therefore, this treatment could not influence the results of the humoral responses of the patients who received it.

### Cellular responses

Cellular responses were assessed by Interferon-Gamma Release Immuno-Assays (IGRAs). Blood samples for IGRAs were collected between 8am and 12pm in tubes containing lithium heparinate. After receipt in the laboratory and within eight hours from blood collection, immune cells in whole blood were stimulated with immune agents. To measure IFN-γ levels produced after nonspecific stimulation of both innate and adaptative immune cells, we used the QuantiFERON^®^-Monitor test (Qiagen) in which one millilitre of whole blood was collected in tubes containing immune agents that mimic pathogen-associated molecular patterns (R848 as TLR7/8 agonist and anti-CD3 as T-cell stimulant). To measure IFN-γ levels produced by SARS-CoV-2-specific T cells, we used the QuantiFERON^®^ SARS- CoV-2 test (Qiagen) in which one millilitre of whole blood was collected in tubes containing a mixture of SARS-CoV-2 peptides. Stimulated blood samples were incubated for 18 ± 2 hours at 37°C and centrifugated at 2000-3000 x g for 15 minutes to harvest the plasmas. Plasmas were then stored at -80°C until analysis and freeze-thaw cycles were minimized to preserve the quality of the samples. Plasma IFN-γ levels after nonspecific and specific stimulations were measured by ELISA.

### Statistics

Data are presented as mean and standard deviation for quantitative variables with parametric distribution, as median and interquartile range (IQR) [25e percentile; 75e percentile] for quantitative variables with non-parametric distribution, or as numbers and percentages for qualitative variables. The Shapiro-Wilk normality test was used to verify the distribution of data. Comparisons were performed using the unpaired two-sided Student’s t-test or Wilcoxon-Mann-Whitney U test according to data distribution for quantitative variables, and the Fisher’s exact test for qualitative variables. Multiple stepwise regression analysis was used to explore factors associated with oxygen requirement. Receiver Operating Characteristic (ROC) curve was used to define an IFN-γ threshold after specific stimulation below which patients would be at risk of severe COVID-19 defined as the need for oxygen therapy. Patients with an IFN-γ response above this threshold were considered to have a protective cellular response. Patients with anti-spike IgG level greater than 264 BAU/mL (21) or who received casirivimab/indevimab (i.e., preventive anti-SARS-CoV-2 monoclonal antibodies) were considered to have a protective humoral response. The associations between humoral and cellular responses were compared using Spearman rank correlation coefficient. Reverse Kaplan-Meier curves and LogRank test were used to compare the probability of oxygen therapy at inclusion based on humoral and cellular responses. Statistical analyses were performed using GraphPad Prism 8 (GraphPad Software, Inc., San Diego, CA) and StatView for Windows, version 5.0 (SAS Institute Inc.). All comparisons were two-tailed, and the differences were considered significant when p value< 0.05.

## Results

### Characteristics of the population and outcomes

**Figure 1:**
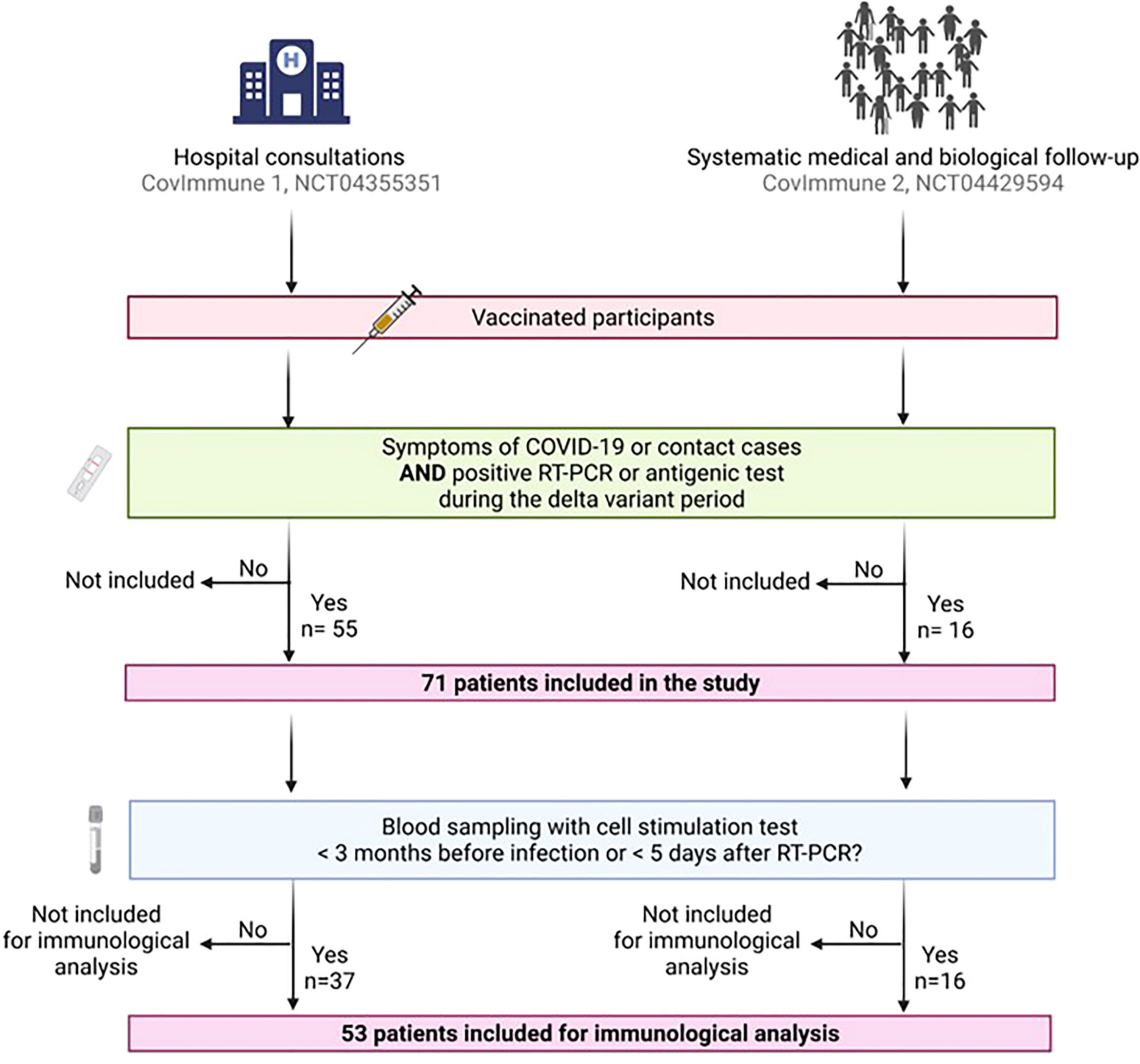
Flow chart showing participants inclusion. The participants were included from two cohorts: **(i)** patients recruited during a hospital consultation following COVID-19 symptoms, or as contact of a diagnosed COVID-19 case (CovImmune 1 study, NCT04355351); **(ii)** participants monitored periodically since July 2020 as part of an epidemiological study in the context of COVID-19 (CovImmune 2 study, NCT04429594) and developing a SARS-CoV- 2 infection. Patients were eligible for inclusion if they received a complete vaccination regimen, and they developed a SARS-CoV-2 infection in the aftermath of the vaccination and during the Delta variant period. Of these 71 patients, 53 could be included for immunological analysis because they had the necessary blood tests at the time of inclusion. COVID-19, coronavirus disease 2019; RT-PCR, reverse transcription-polymerase chain reaction. Created with BioRender.com.

A total of 71 patients were included in this ancillary study, divided into 55 patients from the CovImmune 1 cohort, and 16 participants from the CovImmune 2 cohort. (**Figure 1**). The main clinical characteristics of this population were reported **Table S1** (see supplemental data), left column. Within this population, a subset of 53 patients had available immunological samples and was subsequently analysed. **Table 1** describes the main characteristics of patients enrolled in the immunological analysis. A slight feminine predominance was observed (57%), the median age was 58. Nearly half of the participants were overweight (47%) and a third of them had more than one comorbidity. Almost all of them had been vaccinated with an mRNA vaccine (88%) and 32% had a third (booster) dose. Passive immunisation by anti-SARS-CoV-2 monoclonal antibodies (imdevimab/casirivimab) had been previously administered in 15 cases (28%). The median time between the last vaccine dose and the onset of COVID-19 symptoms (or positive test for asymptomatic cases) was 132 days. Ten patients were hospitalised, 12 required oxygen therapy, 3 were admitted in intensive care unit (ICU) and 3 died from refractory respiratory failure.

**Table 1:** Characteristics of patients and their post vaccination immune response according to oxygen requirement.

	Total n=53	Oxygen support n=12	No oxygen support n=41	p-value
Baseline characteristics				
Age, years	58 [42-72]	75.0 [63.8-78.5]	53.0 [41-62]	**0.013**
Male gender, n (%)	23 (43%)	8 (67%)	15 (29%)	**0.039**
Overweight (BMI > 25 kg/m²)	25 (47%)	9 (75%)	16 (36%)	**0.047**
More than one comorbidity*, n (%)	16 (30%)	9 (75%)	7 (17%)	**<0.001**
Hypertension	11 (19%)	6 (50%)	5 (12%)	**<0.001**
Diabetes	5 (9%)	4 (33%)	1 (2%)	**0.007**
Active cancer of hemopathy	7(13%)	5 (42%)	2 (5%)	**0.004**
SARS-CoV-2 vaccination				
Type of vaccine				0.146
mRNA vaccine, n (%)	48 (88%)	9 (75%)	38 (93%)	0.121
Adenovirus vaccine, n (%)	3 (6%)	2 (17%)	1 (2%)	0.125
Combined, n (%)	3 (6%)	1 (8%)	2 (5%)	0.545
Delay since last dose (days)	132 [44-188]	120 [58.5-148-8]	142 [37-201]	0.808
Delay since last dose > 3 months, n (%)	20 (38%)	8 (67%)	12 (57%)	0.719
Booster dose, n (%)	17 (32%)	2 (16%)	15 (36%)	0.296
SARS-CoV-2 breakthrough infection				
Presence of symptoms at COVID-19 diagnosis	48 (91%)	12 (100%)	36 (87%)	0.879
Time from blood collection to onset of symptoms, days	4 [2-7]	7 [4-13]	4 [2-5]	**0.008**
Humoral and cellular immune responses				
Anti-SARS-Cov-2 IgG Ab (BAU/mL)	275.9 [28.9-1664.0]	473.0 [168.3-1743]	251.8 [13.1-1459]	0.541
Inhibition of the Delta variant (%)	68 [18-99]	66 [21-99]	68 [13-95]	0.855
Specific CD4^+^ CD8^+^ response (IFN-γ, IU/mL)	0.35 [0.12-0.76]	0.11 [0.02-0.27]	0.53 [0.14-0.95]	**0.021**
Nonspecific cellular response (IFN-γ, IU/mL)	84 [17-349]	9 [4-57]	123 [24-407]	**0.004**
Treatments and outcomes				
Preventive anti-SARS-CoV-2 mAb, n (%)	15 (28%)	2 (17%)	13 (31%)	0.472
Hospitalization, n (%)	10 (19%)	10 (83%)	0 (0%)	**<0.001**
Corticosteroids, n (%)	12 (23%)	8 (67%)	3 (7%)	**<0.001**
Tocilizumab, n (%)	3 (6%)	3 (25%)	0 (0%)	**0.009**
ICU admission, n (%)	3 (6%)	3 (25%)	0 (0%)	**0.009**
Death at 28 days, n (%)	3 (6%)	3 (25%)	0 (0%)	**0.009**

The number (and percentage) are indicated for categorical variables, median (and interquartile range) for continuous variable. Comparisons were performed using Wilcoxon-Mann-Whitney U test for quantitative variables, and the Fisher’s exact test for qualitative variables. Significant associations are in bold.

* comorbidities: cardiovascular disease, obesity, respiratory diseases, haematological or solid organ malignancies, Ab, antibodies; BAU, binding antibody units; BMI, body mass index; IFN-γ, interferon-gamma; mAb, monoclonal antibodies; mRNA, messenger RNA vaccine; NA, not applicable.

### Factors associated with oxygen requirement

As shown in previous studies (27–30), unadjusted analysis confirmed that the risk of severe COVID-19, (defined here as the need of oxygen therapy) was significantly higher with increased age (p=0.002), high body mass index (BMI) (p<0.001) and comorbidities such as hypertension (p<0.001) and type 2 diabetes (p=0.002) (**Table S1**). In this cohort, mRNA vaccination seemed more likely to be associated with a favourable outcome (p=0.041), as well as having received a booster dose (p=0.024). However, post-vaccination delay was not associated with a better prognosis. Patients who had previously received the administration of preventive anti-SARS-CoV-2 monoclonal antibodies (casirivimab/imdevimab) had a better prognosis than others (p=0.02). All variables associated with oxygen requirement and reaching a p<0.2 (**Table S1**) were included in the backward stepwise regression analysis. In the final model, two independent variables were associated with oxygen requirement: being older than 65 years (odds ratio (OR), 5.1 [95% CI, 1.5-17.5], p=0.008) and having at least one comorbidity (OR, 4.5 [95% CI, 1.1-17.5].

### Humoral and cellular post-vaccination protective responses to severe COVID-19

**Figure 2:**
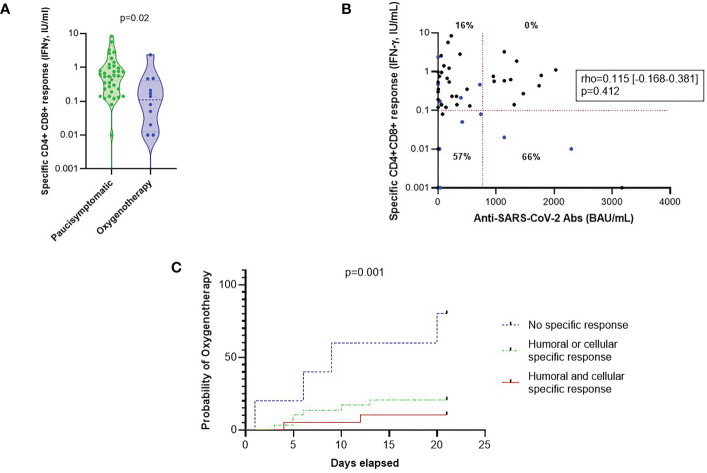
Specific cellular and humoral responses post-vaccination and the risk of oxygen requirement after breakthrough SARS-CoV-2 infection. **(A)** Comparison of CD4^+^ CD8^+^ specific responses according to oxygen requirement. Statistical significance of difference between groups was assessed using Mann-Whitney non-parametric test. **(B)** Correlation between CD4^+^ CD8^+^ specific responses and anti-SARS-CoV-2 antibody titers (BAU/mL). Patients who required oxygen therapy are represented by blue dots, others by black dots. Percentages represent the rates of patients on oxygen therapy in each dial. The level of IFN-γ after specific CD4^+^ and CD8^+^ stimulation required for a protective response is represented by a horizontal red line (i.e. 0.10 IU/mL). The level of anti-SARS-CoV-2 Ab required for a protective response in this cohort is represented by a vertical red line (i.e. 760 BAU/mL). The association between humoral and cellular responses were compared using Spearman rank correlation coefficient. **(C)** Oxygen-free survival rate based on humoral and cellular responses. Patients with an anti-SARS-CoV-2 antibody level greater than 264 BAU/mL or who received preventive anti-SARS-CoV-2 mAb were considered to have a good humoral response. Patients with IFN-γ level after specific stimulation greater than 0.10 IU/mL were considered to have a good cellular response. The IFN-γ threshold of 0.10 IU/mL was determined by ROC curve with which we made the choice to favor specificity (sensitivity 50% and specificity 85%). A reverse Kaplan-Meier curve and LogRank test analysis were made to study these data. IFN, interferon; mAb, monoclonal antibodies; ROC, receiver-operating characteristics.

Among the 71 patients included, 53 of them had an available blood sample with cell stimulation within three months prior to infection or within five days after the positive RT-PCR or antigen test (**Figure 1**). Of these 53 patients, 12 required oxygen therapy during their illness. In this subgroup, we found no differences in the type of vaccine, number of vaccine doses, anti-SARS-CoV-2 IgG antibody level, and percentage of Delta variant neutralization according to patient outcome. However, we found that patients who did not require oxygen therapy had significantly higher SARS-CoV-2-specific (p=0.021) and nonspecific (p=0.004) cellular responses before or at the onset of infection than those who progressed to a severe form (**Table 1**; **Figure 2A**). Vaccine protection (defined by the absence of a severe form requiring oxygen therapy) was the most efficient in patients who had developed both a sufficient level of anti-SARS-CoV-2 antibodies (>760 BAU/ml) and a strong specific cellular response above (IFN-γ level after specific stimulation greater than 0.10 IU/mL) (**Figure 2B**). Surprisingly, we did not find any correlation between specific cellular response and anti-SARS-CoV-2 antibody levels (rho=0.115 [CI 95%, -0.168-0.381], p=0.412; **Figure 2B**), but we found a positive correlation between specific cellular response and Delta variant neutralization (rho=0.281 [CI 95%, 0.003-0.518], p=0.042) (**Figure S1**). To note and as expected, we found a strong positive correlation between anti-SARS-CoV-2 antibody levels and Delta variant neutralization (rho=0.709 [CI 95%, 0.537-0.825], p<0.001) (**Figure S1**).

We then studied the time from diagnosis of COVID-19 to initiation of oxygen therapy according to humoral (including preventive monoclonal antibody therapy or anti-spike IgG level greater than 264 BAU/mL) and cellular responses (defining by IFN-γ level after specific stimulation greater than 0.10 IU/mL). We found that patients with high specific immune responses (post-vaccination and/or post-treatment with monoclonal antibodies) had a significantly lower probability of oxygen therapy (p=0.001) (**Figure 2C**).

## Discussion

In this ancillary prospective study, we investigated the humoral and cellular immune responses of fully vaccinated individuals who experienced breakthrough infection due to the SARS-CoV-2 Delta variant and correlated these measures with the severity of COVID-19. We found that an effective humoral response, as previously defined (22) or administration of preventive anti-SARS-CoV-2 monoclonal antibodies, was not sufficient alone to induce an effective immune protection against severe breakthrough infection, which also required an effective cell-mediated immunity to SARS-CoV-2. We found no correlation between anti-SARS-CoV-2 antibody titers and cellular response in this cohort, but a modest correlation between neutralization titers and the extent of cell-mediated immunity to SARS- CoV-2, confirming previous data obtained in specific patient populations (31–33). This result highlights the importance of collaboration between B and T cells to improve the affinity of antibodies against their antigenic target, allowing the initiation of somatic mutations in the variable region.

Clinical data of our cohort are consistent with already known risk factors of severe COVID-19 such as age, comorbidities, and absence of preventive specific monoclonal antibodies in the unadjusted analysis, confirmed for the first two in the multivariable analysis. The limited size of our cohort does not allow to get more specific clinical insights.

The choice of defining a full vaccination as two vaccine doses could be discussed, since a third and even a fourth dose (“boosters”) are now mainly recommended, in a timed manner (https://www.cdc.gov/coronavirus/2019-ncov/vaccines/stay-up-to-date.html). However, only 20% of the cohort was more than 6 months away from the last vaccine dose, and large studies have demonstrated that a two doses “full vaccination” is highly effective to prevent hospitalization (34). But more to the point, the purpose of our research is to correlate the level of both measured humoral and cellular post- vaccine immunity to the risk of severe COVID-19, rather than assessing the vaccine efficacy itself (even if our results showed that patients requiring no oxygen received more frequently a third dose). So, our findings could be used whatever the previous immunization mode.

Another point to be addressed is the vaccine-induced immune response regarding the type of variant. Emergence of Omicron variant was associated to an incomplete escape to mRNA vaccine-induced neutralizing antibodies, assessed as less than 30 times neutralizing activity of mRNA vaccine sera (compared to 614D strain reference), which it is not the case with the Delta variant with a limited 2.6 decrease of neutralizing activity (35, 36). Thus, our choice to restrict the study period to the Delta wave in our area, although reducing the number of included patients, strengthen our data consistency.

Further studies are needed to understand the factors that stimulate one or the other of the immune responses, i.e., humoral and/or cellular immune responses, or on the contrary lead to a failed immune response, as may be the case for the demographic characteristics and comorbidities inherent to each individual (37). The risk factors for developing a severe COVID-19 are here the same as those previously described, i.e., age, BMI, at least one comorbidity (27–30), and also protective factors, i.e. vaccination and the administration of therapeutic monoclonal antibodies. Although several studies have reported a decrease in vaccine effectiveness with increasing time since vaccination (38, 39), we did not find this result probably due to a too small sample. A more comprehensive tool to simply assess both humoral and cellular post-vaccination responses could allow better targeting of patients remaining at risk of severe COVID-19 despite vaccination and who could benefit from a reinforcement of preventive strategies, or even a modification of the vaccination schedule (e.g., mode of administration, additional dose, type of vaccine…).

The originality of this study on vaccine breakthrough infections lies in the prospective follow-up and immunological analyses prior to the aggravation of the COVID-19. As recently reported, the relation of type II IFN release to the risk of negative outcome is suggested here (40).

However, this study has several limitations. First, although the results are significant, the sample size is small, which limits the magnitude of the findings. Indeed, the small sample size results in a heterogeneous cohort, particularly in terms of the type of vaccination received (85% mRNA vaccine, 11% adenovirus vaccine and 4% combination) and the time between blood collection and onset of COVID-19 symptoms. Prospective studies on larger cohorts need to be conducted to confirm these results. Second, the cohort was not representative of the population with many comorbid patients and deaths following infection. This could be explained by the mode of recruitment (hospital consultation and not in city offices) and by the aging demography of our region. Finally, the cutoff value applied to define positive responses by the IFN-γ test after specific stimulation needs to be replicated in another prospective cohort.

In conclusion, we found that the severity of breakthrough infections with COVID-19 Delta variant in vaccinated individuals can be predicted based on their humoral and specific cellular post vaccine responses measured at the onset of the infection. The levels of these responses do not seem to be correlated with one another at the individual level, however, the conjunction of both a strong humoral and a specific cellular response are associated with a favourable outcome in case of a breakthrough infection. This brings insights for the future development of biomarkers able to discriminate vaccinated patients at risk of developing serious breakthrough infections. This approach deserves larger clinical studies to confirm our data, including infection by other variants, in vaccinated patients. If confirmed, our data open the door to a proposal of vaccine passport, helping to schedule additional boosters more appropriately.

## Data availability statement

The raw data supporting the conclusions of this article will be made available by the authors, without undue reservation.

## Ethics statement

The studies involving human participants were reviewed and approved by COMITE de PROTECTION des PERSONNES SUD-OUEST ET OUTRE-MER II. The patients/participants provided their written informed consent to participate in this study.

## Author contributions

The original concept and design of the study was made by BS-P, MiC, MB, MaC. MiC, MB, MaC and BS-P participated in the acquisition of the data. VB, DG and MaC carried out experiments. MaC, BS-P, MiC, and MB analysed and interpreted the data. MaC, BS-P, MiC, EC, MB and JA performed the statistical analyses. MaC, MB, MiC, and BS-P drafted the manuscript. All authors contributed to the article and approved the submitted version

## Supplementary material

Supplementary Figure 1 Specific cellular and humoral responses post-vaccination. (A) Correlation between CD4+ CD8+ specific responses and Delta variant neutralization. The association was compared using Spearman rank correlation coefficient. (B) Correlation between anti-SARS-CoV-2 antibody levels and Delta variant neutralization. The association was compared using Spearman rank correlation coefficient.
