# Supplementary figures and images for: Breakthrough infections due to SARS-CoV-2 Delta variant: relation to humoral and cellular vaccine responses
Source: Front Immunol. 2023 Mar 30;14:1145652. doi: 10.3389/fimmu.2023.1145652 (PMC10101330; doi:10.3389/fimmu.2023.1145652)

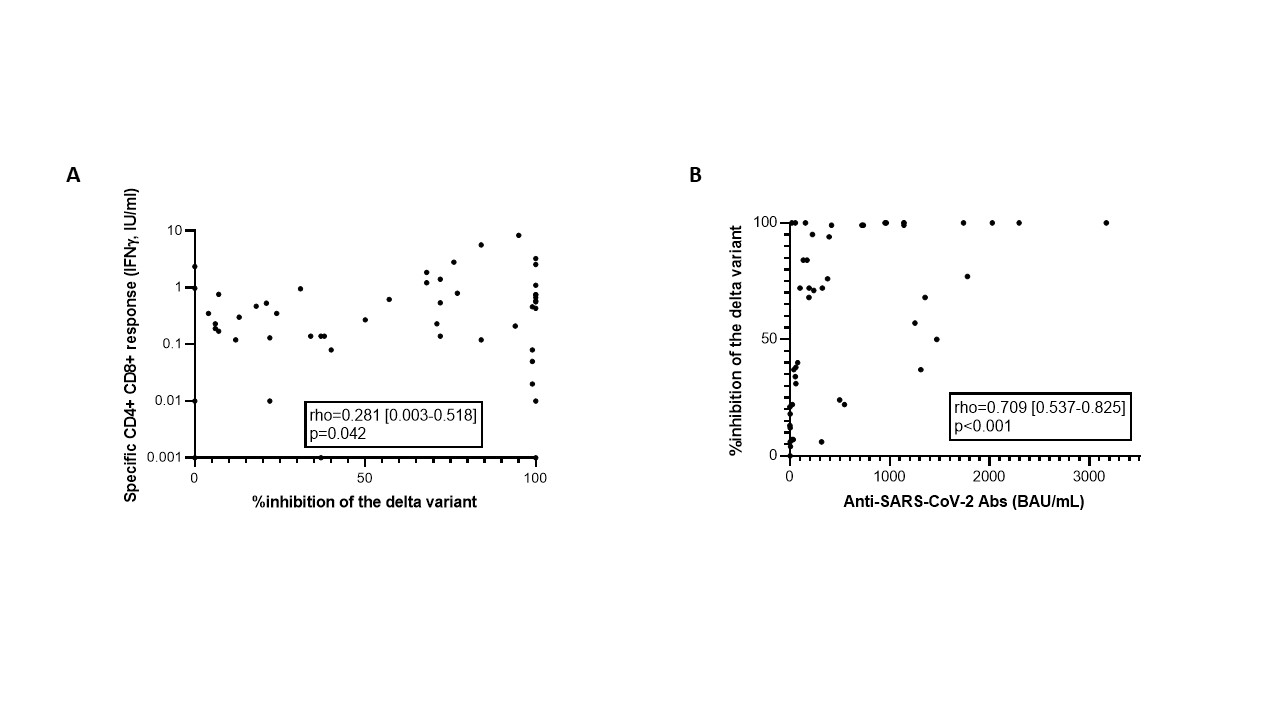

Supplement: Supplementary Figure 1 — Specific cellular and humoral responses post-vaccination. (A) Correlation between CD4+ CD8+ specific responses and Delta variant neutralization. The association was compared using Spearman rank correlation coefficient. (B) Correlation between anti-SARS-CoV-2 antibody levels and Delta variant neutralization. The association was compared using Spearman rank correlation coefficient. [file Image_1.jpeg]
