# Supplementary material for: Breakthrough infections due to SARS-CoV-2 Delta variant: relation to humoral and cellular vaccine responses
Source: Front Immunol. 2023 Mar 30;14:1145652. doi: 10.3389/fimmu.2023.1145652 (PMC10101330; doi:10.3389/fimmu.2023.1145652)
Supplement: Supplementary file 2 [file Table_1.docx]

## Table S1: Cohort data and comparisons regarding oxygen requirement

|  | **Full cohort**  **n=71** | **Oxygen support**  **n=29** | **No oxygen support**  **n=42** |  | |
| --- | --- | --- | --- | --- | --- |
|  |  |  |  | **p-value** | **OR [95% CI]** |
| **Baseline characteristics** |  |  |  |  |  |
| Age, years | 58 [42-72] | 72 [51-78] | 53 [40-61] | **0.002** | - |
| Age > 65 years, n (%) | 26 | 18 | 8 | **<0.001** | 6.73 [2.11 ; 23.58] |
| Male gender, n (%) | 30 (41%) | 15 (52%) | 15 (36%) | 0.225 | 1.91 [0.66 ; 5.64] |
| BMI, kg/m² | 25  [22-30] | 28 [25-32] ^a^ | 24 [21-26] | **<0.001** | - |
| Overweight (BMI ≥ 25 kg/m²) | 37 (53%) | 21(77%) ^a^ | 16 (38%) | **0.001** | 5.535 [1.702 ; 20.560] |
| At least one comorbidity, n (%) | 42 (59%) | 25 (86%) | 17 (40%) | **<0.001** | 8.886 [2.458 ; 41.548] |
| Type 2 diabetes, n (%) | 9 (13%) | 8 (28%) | 1 (2%) | **0.002** | 16.579 [1.992 ; 780.686] |
| Hypertension, n (%) | 20 (28%) | 15 (52%) | 5 (12%) | **<0.001** | 8.893 [2.447 ; 38.320] |
| Ongoing cancer or hemopathy, n (%) | 8 (11%) | 4 (14%) | 4 (10%) | 0.702 | 1.640 [0.277 ; 9.729] |
| Transplant, n (%) | 4 (6%) | 0 | 4 (9%) | 0.139 | NC |
| COPD or Asthma | 10 (14%) | 6 (21%) | 4 (10%) | 0.297 | 2.446 [0.517 ; 13.101] |
| Cardiovascular diseases^†^ | 23 (32%) | 20 (69%) | 3 (7%) | **<0,001** | 26.982 [6.215 ; 172.775] |
| Preventive anti-SARS-CoV-2 mAb, n (%) | 15 (21%) | 2 (7%) | 13 (31%) | **0.0181** | NC |
| **SARS-CoV-2 vaccination** |  |  |  |  |  |
| *Type of vaccine* |  |  |  |  |  |
| mRNA vaccine, n (%) | 60 (85%) | 21 (72%) | 39 (93%) | **0.041** | 0.206 [0.031 ; 0.976] |
| Adenovirus vaccine, n (%) | 8 (11%) | 7 (24%) | 1 (2%) | 0.006 | 13.05 [1.51 ; 112.98] |
| Combined, n (%) | 3 (4%) | 1 (3%) | 2 (5%) | 1 | 0.699 [0.011 ; 14] |
| Delay since last dose (days) | 120 [54;171] | 120 [66-157] ^b^ | 112 [39-200] ^c^ | 0.937 | - |
| Delay since last dose > 3 months, n (%) | 43 (61%) | 20 (72%) ^b^ | 13 (60%) ^c^ | 0.385 | 1.711 [0.452 ; 6.638] |
| Third (Booster) dose, n (%) | 18 (25%) | 3 (10%) | 15 (36%) | **0.024** | 0.212 [0.035 ; 0.875] |

The number (and percentage) are indicated for categorical variables, median (and interquartile range) for continuous variable. Comparisons were performed using the Wilcoxon-Mann-Whitney U test for quantitative variables, and the Fisher’s exact test for qualitative variables. Significative associations are in bold.

† cardiovascular diseases: coronary heart disease, peripheral arterial disease, and stroke.

^a^ two missing data; ^b^ one missing data; ^c^19 missing data;

BMI, body mass index; CI, confidence interval; COPD, chronic obstructive pulmonary disease; F, female; M, male; mAb, monoclonal antibodies; mRNA, messenger RNA vaccine; NA, not applicable; SpO2, percutaneous oxygen saturation; NC, non-calculable.
